# Screening of the Skin-Regenerative Potential of Antimicrobial Peptides: Clavanin A, Clavanin-MO, and Mastoparan-MO

**DOI:** 10.3390/ijms25136851

**Published:** 2024-06-22

**Authors:** Thuany Alencar-Silva, Rubén D Díaz-Martín, Mickelly Sousa dos Santos, Rivaldo Varejão Pasqual Saraiva, Michel Lopes Leite, Maria Tereza de Oliveira Rodrigues, Robert Pogue, Rosângela Andrade, Fabrício Falconi Costa, Nicolau Brito, Simoni Campos Dias, Juliana Lott Carvalho

**Affiliations:** 1 Programa de Pós-Graduação em Ciências Genômicas e Biotecnologia, Universidade Católica de Brasília, Brasília 71966-900, Brazil; thuanyalencar@gmail.com (T.A.-S.); rubendario025@gmail.com (R.D.D.-M.); mickelly.123@gmail.com (M.S.d.S.); rivaldo.vps8@gmail.com (R.V.P.S.); mariaatereza96@gmail.com (M.T.d.O.R.); redward@p.ucb.br (R.P.); rosangelavand@gmail.com (R.A.); fcosta@genomicenterprise.com (F.F.C.); si.camposdias@gmail.com (S.C.D.); 2 Departamento de Biologia Molecular, Instituto de Ciências Biológicas, Universidade de Brasília, Brasília 70910-900, Brazil; michelleitte@gmail.com; 3 Faculdade de Agronomia e Medicina Veterinária, Universidade de Brasília, Brasília 71966-700, Brazil; nicolaubrt@gmail.com; 4 Programa de Pós-Graduação em Biologia Animal, Universidade de Brasília, Brasília 71966-700, Brazil; 5 Laboratório Interdisciplinar de Biociências, Faculdade de Medicina, Universidade de Brasília, Brasília 70910-900, Brazil

**Keywords:** antimicrobial peptides, Clavanin A, Clavanin-MO, Mastoparan-MO, skin regeneration

## Abstract

Skin wound healing is coordinated by a delicate balance between proinflammatory and anti-inflammatory responses, which can be affected by opportunistic pathogens and metabolic or vascular diseases. Several antimicrobial peptides (AMPs) possess immunomodulatory properties, suggesting their potential to support skin wound healing. Here, we evaluated the proregenerative activity of three recently described AMPs (Clavanin A, Clavanin-MO, and Mastoparan-MO). Human primary dermal fibroblasts (hFibs) were used to determine peptide toxicity and their capacity to induce cell proliferation and migration. Furthermore, mRNA analysis was used to investigate the modulation of genes associated with skin regeneration. Subsequently, the regenerative potential of the peptides was further confirmed using an ex vivo organotypic model of human skin (hOSEC)-based lesion. Our results indicate that the three molecules evaluated in this study have regenerative potential at nontoxic doses (i.e., 200 μM for Clavanin-A and Clavanin-MO, and 6.25 μM for Mastoparan-MO). At these concentrations, all peptides promoted the proliferation and migration of hFibs during in vitro assays. Such processes were accompanied by gene expression signatures related to skin regenerative processes, including significantly higher *KI67*, *HAS2* and *CXCR4* mRNA levels induced by Clavanin A and Mastoparan-MO. Such findings translated into significantly accelerated wound healing promoted by both Clavanin A and Mastoparan-MO in hOSEC-based lesions. Overall, the data demonstrate the proregenerative properties of these peptides using human experimental skin models, with Mastoparan-MO and Clavanin A showing much greater potential for inducing wound healing compared to Clavanin-MO.

## 1. Introduction

Normal wound healing involves coordination between the immune response and the processes of cellular migration, proliferation, matrix deposition, and tissue remodeling [1]. During skin wound healing, an excessive inflammatory response induced by opportunistic pathogens, and metabolic, or chronic diseases can lead to nonhealing wounds [2,3]. For instance, an increase in the proinflammatory response, resulting from the deregulation of several key proinflammatory cytokines, such as IL-1β and tumor necrosis factor–α (TNFα), prolongs the inflammatory phase, and has been related to elevated activity of metalloproteinases that impair cell migration and proliferation process, thus delaying the wound repair [3,4]. This deterioration in healing conditions and the presence of nonhealing wounds increases treatment costs, causes aesthetic damage, or even puts the lives of patients at risk, which makes it necessary to search for new therapeutic options that favor skin tissue regeneration [1,4].

In the search for new proregenerative treatments, antimicrobial peptides (AMPs) arise as a group of molecules that present a broad spectrum of antimicrobial activity, accompanied by immunomodulatory and regenerative properties that promote cell proliferation, and angiogenesis, which favors the wound healing process [5,6]. The AMPs Clavanin A (VFQFLGKIIHHVGNFVHGFSHVF-NH2) and its synthetic derivative Clavanin-MO (FLPIIVFQFLGKIIHHVGNFVHGFSHVF-NH2) present strong activity against both Gram-negative and Gram-positive bacteria and can modify the expression of immune system components that regulate the inflammatory response [7,8,9]. Clavanin-MO can modulate innate immunity by stimulating leukocyte recruitment and the production of immune mediators such as GM-CSF, IFN-γ, and MCP-1, favoring an increase in the levels of anti-inflammatory cytokines (IL-10) and repressing the expression of proinflammatory cytokines (IL-12 and TNF-α), which suppresses the damage caused by an excessive inflammatory response [9]. Likewise, the peptide Mastoparan-MO (FLPIIINLKALAAALAKKIL), a less toxic synthetic variant of the natural AMP Mastoparan-L, can increase leukocyte migration while suppressing the expression of proinflammatory factors such as TNF-α, and IL-6. These immunomodulatory properties enhance the immune responses required to eliminate infections and reduce the damage caused by an excessive inflammatory response [10,11].

In the present study, we examined the ability of three antimicrobial peptides (Clavanin A, Clavanin-MO, and Mastoparan-MO,) to stimulate cell proliferation, and migration, as well as encourage the activation of genes associated with tissue regeneration in a model of primary human dermal fibroblasts (hFibs). Likewise, using an organotypic human ex vivo skin model (hOSEC), we analyzed the possible induction of wound healing generated by these antimicrobial peptides.

Our results indicate that treatment with Clavanin A, Clavanin-MO, or Mastoparan-MO peptides did not generate changes in the cellular viability of primary skin cells, suggesting low cytotoxicity of these molecules. Likewise, treatment with these AMPs can increase cell proliferation, migratory capabilities, and the induction of gene expression related to tissue repair processes in hFibs. Finally, using an ex vivo human skin culture system, we determined that Clavanin-MO and Mastoparan-MO can potentially facilitate wound healing. These results suggest that these antimicrobial and immunomodulatory peptides have skin-regenerative potential, constituting an exciting alternative for the development of further treatments for skin injuries.

## 2. Results

### 2.1. Cytotoxicity Screening of AMPs

**Figure 1:**
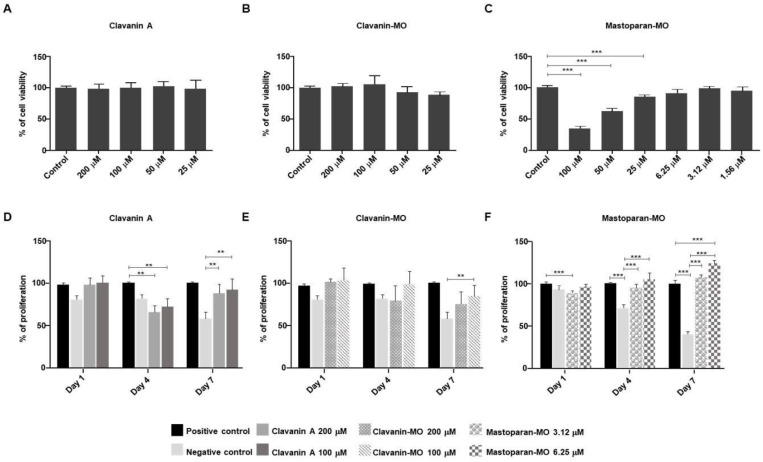
Cytotoxicity screening of AMPs. Cytotoxic potential of Clavanin A and Clavanin-MO at 200, 100, 50, and 25 μM ((**A**,**B**) respectively), and Mastoparan-MO at 100, 50, 25, 6.25, 3.12, and 1.56 μM (**C**), was tested in human primary dermal fibroblasts (hFibs). The mean ± SD of three independent experiments is presented. Effects on cell proliferation of Clavanin A and Clavanin-MO at 200 or 100 μM ((**D**,**E**), respectively) and Mastoparan-MO at 6.25 or 3.12 μM (**F**), were determined at 1, 4, and 7 days by MTT assay. The mean, along with the standard deviation, is shown for three independent experiments. In both cases, the statistical analysis was carried out through one-way ANOVA and Tukey post hoc tests. Asterisks indicate significant differences (** *p* < 0.01; *** *p* < 0.001) relative to the control.

We analyzed the cytotoxic potential on skin cells of three well-characterized antimicrobial peptides (Table 1). At 24 h of exposure, the peptides Clavanin A and Clavanin-MO did not produce any significant change in cell viability compared to control when the cells were exposed to doses as high as 200 μM (Figure 1A,B). However, Mastoparan-MO induced a significant and progressive reduction in cell viability, observed starting from 25 μM (*p* < 0.001), and reaching more than 50% at 100 μM (*p* < 0.001) (Figure 1C).

This peptide, however, did not generate a significant change in cell viability when the cells were exposed to concentrations below 25 μM. As cytotoxicity controls, we evaluated the peptides Polybia-MPII and EcDBS1R6, which induced a significant reduction in cell viability (*p* < 0.05) at all concentrations tested (Figure S1). Based on these results, for further experiments, we used Clavanin A and Clavanin-MO at 100 and 200 μM, while Mastoparan-MO was used at 6.25 and 3.12 μM.

### 2.2. Proliferative Potential

**Figure 2:**
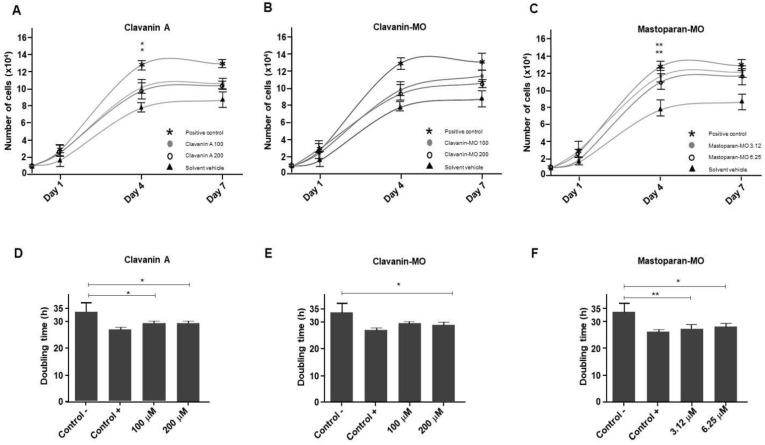
Analysis of proliferation kinetics. Human primary dermal fibroblast (hFibs) were incubated with Clavanin A ((**A**,**D**); 200 or 100 μM), Clavanin-MO ((**B**,**E**); 200 or 100 μM), or Mastoparan-MO ((**C**,**F**); 6.25 or 3.12 µM). Proliferation kinetics (**A**–**C**) were determined at 1, 4, and 7 days by counting the total number of cells. To determine the proliferative potential of the cells, the population doubling time (PDT) was calculated at 4 days (**D**–**F**) of peptide treatment. The statistical analysis was carried out by means of a one-way ANOVA and Tukey post hoc tests. Significant differences regarding the control are marked with asterisks (* *p* < 0.05; ** *p* < 0.01).

At 7 days of treatment, both Clavanin A (100 μM and 200 μM) and Clavanin-MO (100 μM) were able to induce a significant increase in cell proliferation compared to the control of the solvent vehicle (*p* < 0.01 compared to the untreated control; Figure 1D,E). This trend can also be observed on days 1 and 4 of the treatment with Clavanin-MO at 200 μM. Likewise, the treatment with Mastoparan-MO at 3.12 and 6.25 μM induces a significant increase in cell proliferation (compared to the control of the solvent vehicle) at 4 and 7 days of treatment (*p* < 0.001; Figure 1F). After seven days of treatment, it is possible to observe that the Mastoparan-MO treatment at 6.25 μM can induce a significant increase in cell proliferation compared to the positive control (supplemented with 10% FBS) (*p* < 0.001; Figure 1F). The analysis of the proliferation kinetics (Figure 2A–C) reveals that at 4 days, the treatment with Clavanin A or with Mastoparan-MO at any of the tested concentrations produces a significant increase in cell replication rate compared with the control of the solvent vehicle (Clavanin A at 100 μM or 200 μM, *p* <0.05; Mastoparan-MO at 3.12, *p* < 0.05 and at 6.25 μM, *p* < 0.01). In the same way, the analysis of the proliferative potential (Figure 2D–F) that was carried out by calculating the population doubling time (PDT) shows that except for cells treated with Clavanin-MO at 100 μM, all treatments generated a significant reduction in the population doubling time compared to the vehicle control (Clavanin A at 100, 200 μM, and Clavanin-MO at 200 μM, *p* < 0.05; Mastoparan-MO at 3.12 μM, *p* < 0.01, and Mastoparan-MO at 6.25 μM, *p* < 0.05).

### 2.3. Gene Expression Profile

**Figure 3:**
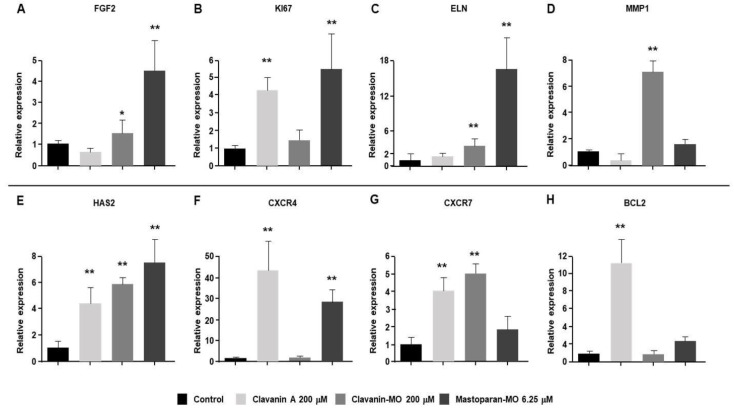
Changes in the expression of proregenerative genes. Relative mRNA expression profile of Human primary dermal fibroblasts (hFibs), that were treated with Clavanin A (200 μM), Clavanin-MO (200 μM), or Mastoparan-MO (6.25 μM), was analyzed. The mRNAs assessed correspond to (**A**) fibroblast growth factor 2 (*FGF2*), (**B**) marker of proliferation Ki-67 (*KI67*), (**C**) elastin (*ELN*), (**D**) matrix metalloproteinase 1 (*MMP1*), (**E**) hyaluronic acid synthase 2 (*HAS2*), (**F**) C-X-C chemokine receptor type 4 (*CXCR4*), (**G**) C-X-C chemokine receptor type 7 (*CXCR7*), and (**H**) B-cell lymphoma 2 protein (*BCL2*). Glyceraldehyde 3-phosphate dehydrogenase (*GAPDH*) was used as a ubiquitous control. Analysis of the mean ± SD of three independent experiments is presented. Statistical analysis was carried out by means of a one-way ANOVA and Tukey post hoc tests. Significant differences regarding the control are marked with asterisks (* *p* < 0.05; ** *p* < 0.01).

Using qRT-PCR, we analyzed potential changes in gene expression of proregenerative factors in cells treated with the peptides (Figure 3). This analysis was conducted with cells treated with the peptides at concentrations of 200 μM for Clavanin A and Clavanin-MO, and 6.25 μM for Mastoparan-MO, as at these concentrations, the peptides showed a significant increase in cell proliferation and a significant reduction in population doubling time (PDT), which suggests a possible proregenerative effect of these molecules. The obtained results revealed that the treatment with Mastoparan-MO generated significant upregulation in expression levels of *FGF2*, *KI67*, *ELN*, *HAS2*, and *CXCR4* transcripts compared to the vehicle control (*p* < 0.01; Figure 3A–C,E,F). Likewise, treatment with Clavanin A can induce a significant upregulation in the expression levels of *KI67*, *HAS2*, *CXCR4*, *CXCR7*, and *BCL2* (*p* < 0.01; Figure 3B,E–H). In contrast, treatment with Clavanin A also generated a trend towards downregulation of *FGF2* and *MMP1* expression (Figure 3A,D). Clavanin-MO treatment promoted a significant upregulation in the expression of *FGF2* (*p* < 0.05 compared to the vehicle control), *ELN*, *MMP1*, *HAS2*, and *CXCR7* (*p* < 0.01 compared to the vehicle control; Figure 3A,C–E,G).

### 2.4. Cellular Migration

**Figure 4:**
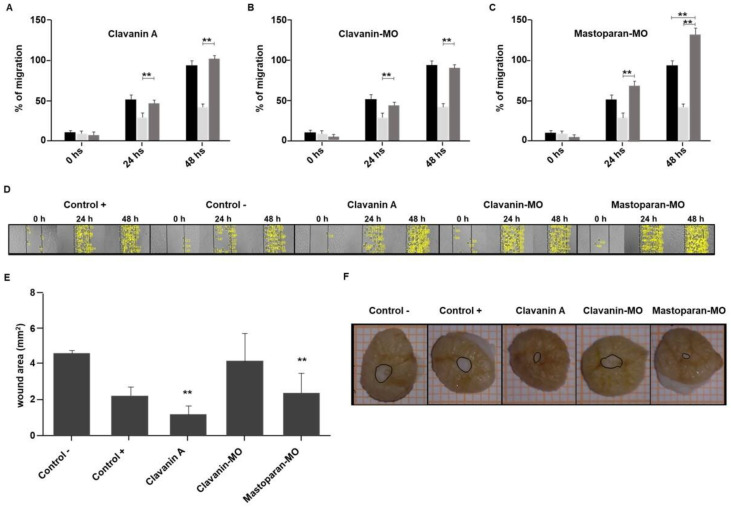
Analysis of fibroblast migration and wound healing in an ex vivo skin model. Human primary dermal fibroblasts (hFibs) were incubated with (**A**) Clavanin A at 200 μM, (**B**) Clavanin-MO at 200 μM, or (**C**) Mastoparan-MO at 6.25 μM, and the cell migration percentage was determined by image analysis. As controls, basal medium supplemented with FBS (positive control, black bars), and basal medium without FSB (negative control, light gray bars). In (**A**–**C**), the dark gray bars correspond to the cells exposed to each of the corresponding peptides. The mean ± SD of the percentage of cells that migrated in three independent experiments at 0, 24, and 48 h is presented. The statistical analysis was carried out by means of one-way ANOVA and Tukey post hoc tests. Significant differences regarding the control are marked with asterisks (** *p* < 0.01). In (**D**), it is possible to observe representative images of hFib migration at 24 and 48 h of peptide treatment (Clavanin A and Clavanin-MO at 200 μM, Mastoparan-MO at 6.25 μM), yellow dots represent the cells that invaded the scratched area. Wound healing assay in human skin explants exposed to: Clavanin A (200 μM), Clavanin-MO (200 μM), or Mastoparan-MO (6.25 μM) (**E**,**F**). Quantification of the area of re-epithelialization and lesion closure in the skin explants at 7 days of treatment was quantified using the ImageJ software (version 1.53 i). The analysis of the mean ± SD of the wound area of three independent experiments is represented (**E**). The statistical analysis was carried out through one-way ANOVA and Tukey post hoc tests. Significant differences regarding the control are marked with asterisks (** *p* < 0.01). In (**F**), it is possible to observe representative images of the closure of the wounds in the human skin explants at 7 days of treatment.

The treatment with both Clavanin A and Clavanin-MO at 200 μM for 24 and 48 h can induce a significant increase in the percentage of cell migration compared to the control of the solvent vehicle without FBS (*p* < 0.01 compared to the untreated control) (Figure 4A,B,D). Likewise, Mastoparan-MO treatment at 6.25 μM (Figure 4C,D) induced a significant increase in cell migration regarding the solvent vehicle control at both 24 and 48 h. At 48 h of treatment, the cells treated with Mastoparan-MO at 6.25 μM show an increase in cell migration that is greater than that observed in the positive control supplemented with FBS (*p* < 0.01; Figure 4C,D).

### 2.5. Wound Healing in hOSEC

The effect of the three AMPs on the wound healing process was analyzed using an ex vivo organotypic model of human skin (hOSEC) (Figure 4E,F). The results indicate that treatment with Clavanin A and Mastoparan-MO at 200 and 6.25 μM, respectively, could induce a significant reduction in the lesion area relative to the negative control of minimal regeneration, treated with the medium supplemented only with FBS (Figure 4E,F) (*p* < 0.01). In contrast, skin explants treated with Clavanin-MO at 200 μM did not show a significant reduction in the lesion area compared to either of the two controls in this experiment.

## 3. Discussion

Growing evidence indicates that several AMPs can generate an immunomodulatory effect that promotes cell attachment, proliferation, and infiltration, thereby facilitating tissue regeneration and wound healing. This suggests that these types of antimicrobial molecules may have the potential to develop proregenerative treatments [12,13,14]. In this work, using both a culture system of primary human fibroblasts (hFibs) and an ex vivo organotypic model of human skin (hOSEC), we analyzed the proregenerative potential of three antimicrobial peptides (Clavanin A, Clavanin-MO, and Mastoparan-MO).

Although Clavanin A and Clavanin-MO have been previously reported to exhibit moderate cytotoxicity against monocyte/macrophage-like cells (EC50 < 50 μM) [15], our results indicate that in hFibs, the treatment with these two calvanins did not generate any significant cytotoxic effect, even at the 200 μM dose. Likewise, even though in RAW 264.7 cells, Mastoparan-MO does not present cytotoxicity at doses of up to 200 μM [10], the absence of cytotoxicity in hFibs could only be observed when the cells were treated with this peptide at concentrations below 25 μM, suggesting that cytotoxicity may depend on the cell type tested [16].

Other peptides that present immunomodulatory properties, such as human beta-defensins 2 and 3 (hBD-2 and hBD-3), can stimulate skin cell proliferation, possibly through the expression of fibroblast growth factor receptor 1 (*FGFR1*), and the enhancement in the phosphorylation of FGFR1, JAK2, and STAT3, which are proregenerative factors that promote wound healing, angiogenesis, and fibroblast activation [12,17,18,19]. Our results indicate that the three AMPs (Clavanin A, Clavanin-MO, and Mastoparan-MO) can induce an increase in the cell proliferation rate of hFibs, which indicates that these peptides, especially Mastoparan-MO, may have properties that facilitate tissue repair through the induction of key genes for cell proliferation such as *FGF2* and *KI67* [20,21].

In this sense, it has also been observed that some antimicrobial peptides, such as the synthetic A-hBD-2 or the LL-37 peptide (the only member of the human cathelicidin family), have the ability to facilitate the wound healing process by stimulating cell migration in both skin cells and mesenchymal stem cells [14,22]. Likewise, Synoeca-MP, an AMP with a broad antimicrobial spectrum, has also been shown to be useful in skin repair when combined with host-defense peptides IDR-1018 [23].

Although the three antimicrobial peptides tested in this study have the potential to induce an increase in cell migration, treatment with Mastoparan-MO can induce a greater increase in the cell migration rate of hFibs. Likewise, upregulation of the *CXCR4* chemokine receptor suggests that the peptides tested can accelerate the migration of epidermal cells through signaling mediated by *CXCL12*, a signaling pathway that has been implicated in the process of wound repair and regeneration [24,25,26].

Other bioactive peptides, such as *Crotalus adamanteus* toxin-II (CaTx-II) and the PR-39 peptide, can induce cell proliferation, which is accompanied by the synthesis of key constituents of the extracellular matrix such as collagen and proteoglycans [27,28]. In this study, we showed that the three peptides tested during this study can generate upregulation of *HAS2*, while Clavanin-MO and Mastoparan-MO induce an increase in the expression of the *ELN* gene, suggesting that these AMPs can facilitate the remodeling of the extracellular matrix (ECM), which is a crucial step in regeneration and wound repair [29,30].

The hOSEC model stands out as an ex vivo system that closely mimics the physiological conditions of human skin. Currently, this alternative model for analyzing the safety and efficacy of various compounds for wound treatment, avoiding the use of animals, is a valuable tool in the validation of compounds for topical and transdermal applications [31]. Ex vivo models that use human skin explants from elective plastic surgery provide a valuable tool to analyze the effect of various molecules in the early stages of the healing process. These models consistently allow for the maintenance of the cell populations present in native skin for at least 7 days of culture. Furthermore, hOSEC models preserve the structure of the extracellular matrix, which contains collagen, elastin, glycosaminoglycans, and other molecules. This enables the results to be more easily extrapolated to the effects of substances on in vivo human skin [32]. hOSEC models have been used to show that specific collagen peptides of fish and porcine origin have the potential to increase collagen and glycosaminoglycan content in the skin, which favors tissue regeneration processes [33]. Similarly, several ex vivo skin models have been used to evaluate the antimicrobial activity and potential skin irritation effects of the AMPs PXL150 and DPK-060. In these models, these AMPs have shown the potential to reduce the colonization of *S. aureus*, highlighting the value of ex vivo models for assessing the effects of peptides on tissue regeneration and skin repair [34,35].

Here, we used lesioned hOSEC models to determine the wound healing potential of Clavanin A, Clavanin-MO, and Mastoparan-MO. Our results showed that Clavanin A and Mastoparan-MO have significantly accelerated wound closure, compared to the non-treated control. Such results are partly explained by the direct effects of both peptides on fibroblasts, which presented higher proliferation and migration upon Clavanin A and Mastoparan-MO treatments. The modulation of genes related to regenerative processes, such as *KI67*, *HAS2* and *CXCR4* might also be involved, since such genes were significantly increased by both peptides. For Clavanin A, the modulation of *CXCR7* and *BCL2* might complement the underlying mechanism of action, while the mechanism of action of Mastoparan-MO involves *FGF2* and *ELN* modulation. Despite promoting fibroblast migration and reducing fibroblast doubling time, Clavanin-MO only promoted a trend towards wound closure acceleration in lesioned hOSEC samples.

The proregenerative potential of various AMPs is associated with their ability to modulate the inflammation process, which is key in the restoration of normal tissue architecture during wound repair [36]. Peptides such as IDR-1018, which exert their action through regulation of the inflammatory response, can induce the repair of skin wounds [13,23,37]. It has been observed that both Clavanin A and Mastoparan-MO are capable of modulating innate immunity by stimulating leukocyte recruitment to the site of infection and production of immune mediators GM-CSF, IFN-γ, and MCP-1 while suppressing excessive inflammatory response by increasing the synthesis of anti-inflammatory cytokines such as IL-10 and repressing the levels of proinflammatory cytokines IL-6, IL-12, and TNF-α. This suggests that the ability of Clavanin A and Mastoparan-MO to modulate inflammation can be related to the proregenerative properties observed during the ex vivo assay [7,8,9,10,11,38].

It has been observed that combining antimicrobial peptides with immunomodulatory peptides can stimulate proregenerative processes in both monolayer models of human skin cells and 3D culture models of skin equivalents, through the modulation of the inflammatory process [23]. Additionally, it has been observed that the combination of various collagen peptides isolated from *Salmo salar* and *Tilapia nilotica* skin, which have an immunomodulatory effect on the innate immune response, has the potential to accelerate wound healing [39]. Clavanin A and Mastoparan-MO share similar properties of promoting fibroblast migration and proliferation, in addition to modulating common transcripts, such as *KI67* and *HAS2*. Nevertheless, the peptides also presented complementary properties, such as the modulation of *CXCR7* and *BCL2* by Clavanin A, and *FGF2* and *ELN* by Mastoparan-MO. It is possible that such peptides, when combined, could have a dual antibacterial, regenerative, and immunomodulatory effect, like that observed in analyses conducted with the combination of platelet-rich plasma with β-lactams, which simultaneously reduce MRSA infection in skin wounds while facilitating the regeneration process in the skin [40].

Another interesting perspective for Clavanin A and Mastoparan-MO peptides as potential molecules that promote tissue regeneration in skin wounds is their incorporation into nanomaterials. This can enhance their stability and activity by protecting them against degradation and controlling their release [41]. Nanoparticles or nanofiber membranes can also improve the solubility of the peptides and provide targeted delivery specificity. In this context, the use of biomaterials based on natural polymers such as collagen, chitosan, and hyaluronic acid offers controlled release aligned with the wound healing process. The effective development of new biomaterials containing these AMPs must consider solubility, stability, and controlled release, as well as the development of biodegradable formulations to promote immunomodulation, reduce toxicity, and improve re-epithelialization, which is crucial for optimal wound healing outcomes [41,42].

## 4. Materials and Methods

### 4.1. Peptide Obtention and Quantification

Synthetic peptides used in this work were provided by Peptide 2.0 Inc. (Chantilly, VA, USA). The molecular mass and purity of all peptides were analyzed by matrix assisted laser desorption/ionization time of flight mass spectrometry (MALDI-ToF MS) on an AutoFlex Speed instrument (BrukerDaltonics, Billerica, MA, USA). Briefly, each peptide was diluted in deionized water, and 1 μL of each solution was mixed with 10 mg/mL α-cyano-4-hydroxycinnamicacid saturated matrix solution, prepared in H2O:ACN:TFA (50:50:0.3, *v*:*v*:*v*). Peptides were plated and dried on a MALDI target plate, and their monoisotopic masses were determined using the reflector mode with external calibration, using the Protein Calibration Standard II for mass spectrometry (Bruker Daltonics, Billerica, MA, USA).

### 4.2. Cell Culture

Human primary dermal fibroblasts (hFibs) were obtained from healthy donors, and provided by CellSeq Solutions (Belo Horizonte, MG, Brazil). The cells were cultured in a controlled environment (5% CO_2_, 37 °C, and 95% humidity). The growth medium employed was DMEM (Gibco, USA), supplemented with 10% *v*/*v*. fetal bovine serum (FBS) (Gibco, Waltham, MA, USA) and 1% *v*/*v*. penicillin/streptomycin solution (1000 U/mL) (Invitrogen, Grand Island, NY, USA).

### 4.3. Peptide Treatments

Cytotoxicity was evaluated in hFibs that were treated with 200, 100, 50, and 25 μM Clavanin A or Clavanin-MO for 48 h. In the case of Mastoparan-MO treatment, the cells were treated at 100, 50, 25, 6.25, 3.12, and 1.56 μM. In all experiments, fresh aliquots of the peptides were prepared in deionized water immediately before use. As cytotoxicity controls, the cells were treated under the same conditions using the peptides Polybia-MPII (INWLKLGKMVIDAL-NH2) and EcDBS1R6 (PMKKLFKLLARIAVKIPVW) at concentrations of 100, 50, 25, and 12.5 μM. Regarding the assessment of cell proliferation, population doubling time, and cell motility, the experimental design includes a positive control group (DMEM plus 10% FBS) and control of the utilized solvent vehicle (basal media without FBS). The treatment with the peptides was carried out in the solvent vehicle. All assays were conducted independently in triplicate.

### 4.4. Cell Viability and Cell Proliferation

Cell viability and proliferation were assessed using an MTT assay kit (Sigma Chemicals Co., St. Louis, MO, USA). Briefly, 1 × 10^4^ cells were seeded in 96-well plates and incubated for 24 h for cell attachment, presenting approximately 30% of cell confluence. This guaranteed that cells would not reach 100% confluency until 7 days, which was the longest period of observation. Cell viability was determined at 48 h of peptide treatment, while cell proliferation was indirectly assessed at 1, 4, and 7 days of treatment. For this, 10 µL per well of MTT solution (5 mg/mL) was added to the cultures, and the plates were incubated for 4 h in the dark. Subsequently, DMSO (200 µL per well) was added and incubated for 30 min. A microplate reader (Bio-Tec PowerWave, Santa Clara, CA, USA) was used to determine the optical density at 595 nm. Reference wavelength was taken at 630 nm.

### 4.5. Proliferation Kinetics and Population Doubling Time

Monitoring of cell proliferation rate was performed in a 6-well plate with a density of 1 × 10^4^ per well. The culture medium was changed every 2 days. The growth curve of cells was obtained by harvesting and counting the number of cells per well at 1, 4, and 7 days of treatment. The population doubling time (PDT) during the logarithmic growth phase was calculated according to Díez et al. (2015), using the following formula [43]:

PDT = Culture Time (in hours)/Population doubling number (PDN)

PDN = Log N/No × 3.31

where N is the number of harvested cells at the end of the growth period, and No is the number of seeded cells.

### 4.6. Cell Migration Assay

The effect of peptides on the migratory capability of hFibs was determined by the wound scratch assay according to Liang et al. (2007) [44]. Briefly, cells were seeded in 6-well plates and cultured until confluence. Scratches were made with 200 μL tips, and cell debris was removed by PBS washing before peptide treatments. Wound closure was photo-documented at time 0 and every 24 h using a Zeiss Primo Vert microscope (Carl Zeiss, Heidelberg, Germany), until the scratch was visually closed by any experimental group. The cells that invaded the scratched area were counted using the ImageJ software (National Institutes of Health, Bethesda, MD, USA). Positive migration control at 48 h was used for data normalization. Samples were analyzed as independent triplicates.

### 4.7. Gene Expression Analysis

Gene expression profiles of peptide-treated cells and controls were evaluated by qRT-PCR after 4 days of treatment. Target gene sequences were obtained from Genbank (https://www.ncbi.nlm.nih.gov/genbank/ (accessed on 15 January 2022)), and the primer sets and probes were designed following the standard criteria defined by the SYBRTM using Primer Express^®^ Software v3.0.1 (Thermo Fisher Scientific, Waltham, MA, USA). Information about the primers used for qRT-PCR analysis is shown in Table S1. Genes assessed were *BCL2*, *CXCR4*, *CXCR7*, Elastin (*EL*), *βFGF*, *Ki67*, *MMP1*, and *VEGF*. The *GAPDH* gene was used as a control. Total RNA isolation was performed using TRIzolTM Reagent (Thermofisher, USA) following the manufacturer’s instructions, and the amount and quality of RNA were determined using a NanoDrop 1000 spectrophotometer (NanoDrop, Wilmington, DE, USA). Reverse transcription was performed using a High-Capacity cDNA Reverse Transcription Kit (Thermofisher, USA). Amplification reactions were performed on StepOne Plus equipment (Applied Biosystems, Waltham, MA, USA) using standardized reagents for real-time PCR (SYBR™ Green Master Mix, Thermofisher, USA) added from primer sets specific to each gene, or using Taqman probes (TaqMan™ Universal PCR Master Mix, Thermofisher, USA). StepOne Software v2.3 was used to determine the Ct values, and the results were analyzed by the 2-ΔΔCT analysis method.

### 4.8. Ex vivo Organotypic Model of Human Skin (hOSEC) Culture and Wound Healing Assay

Skin explants were obtained during routine elective cosmetic surgery performed on healthy patients. Ethical approval of this research was granted by the University of Brasília Research Ethics Committee (protocol number 30175020.0.0000.5558). Tissue samples were collected following informed consent. Explants were washed in phosphate buffer solution (PBS, Thermo Fisher Scientific, USA), supplemented with 1% *v*/*v*. of 10,000 U/mL of penicillin/streptomycin solution (Invitrogen, Grand Island, NY, USA), and the hypodermis was removed. Skin explants were cut with a 6 mm full-thickness punch biopsy, and kept in air–liquid interface at 37 °C, 5% CO_2_, and 95% humidity in DMEM supplemented with 10% *v*/*v*. fetal bovine serum (FBS) (Gibco, USA), and 1% *v*/*v*. of 10,000 U/mL of penicillin/streptomycin solution. The wounds were then created within the punch biopsy (2 mm circular biopsy punch). The lesioned skin fragments were placed in metallic support systems and treated with basal medium supplemented with 10% FBS. Control samples were treated with either PBS (negative control), or fibrin gel (positive control for regeneration), prepared by mixing 2.5 μL of fibrinogen at 40 μg/mL, 7.5 μL of water, and 10 μL of thrombin at 25 U/mL. The experimental group samples were treated with the peptides dissolved in the tissue culture medium. Wound healing was observed for 7 days using a Zeiss Primo Vert microscope (Carl Zeiss, Heidelberg, Germany). The change in the lesion area was determined using the ImageJ software (National Institutes of Health, USA).

### 4.9. Statistical Analysis

The experiments were conducted across a minimum of three separate biological replicates and at least two technical replicates. Data analysis was performed with GraphPad Prism^®^ Software, version 7.02 (San Diego, CA, USA, 2017). A *p*-value of less than 0.05 was considered significant.

## 5. Conclusions

In the present study, we analyzed the proregenerative potential of three antimicrobial peptides (Clavanin A, Clavanin-MO, and Mastoparan-MO). The three peptides analyzed showed relatively low toxicity for hFibs and demonstrated the ability to induce cell proliferation and migration in this kind of cell. Interestingly, the Mastoparan-MO peptide exhibited the greatest proregenerative potential among the three molecules analyzed, inducing cell proliferation and migration events that exceeded those observed in the positive control used in this study. Additionally, our molecular analysis revealed a proreparative gene expression profile induced by treatment with these peptides. The use of an hOSEC model of lesioned skin demonstrated that both Clavanin A and Mastoparan-MO could induce a significant reduction in the lesion area compared to the minimal regeneration control. Taken together, the obtained results suggest that the AMPs used in this study have the potential to facilitate skin regeneration. Thanks to their antibacterial and immunomodulatory properties, these molecules could serve as the basis for developing new pharmacological approaches for treating difficult-to-manage wounds.

## Figures and Tables

**Table 1:** List of antimicrobial peptides used during this study and their immunomodulatory properties.

Name	Immunomodulatory Activity	References
Clavanin A	Clavanin A and Clavanin-MO present anti-inflammatory activities in murine macrophage-like cells stimulated with LPS Clavanin A and Clavanin-MO can increase the production of IL-10 and reduce the expression of proinflammatory IL-12 and TNF-α Clavanin-MO can induce the migration of leukocytes In mice, treatment with Clavanin-MO increased the expression of GM-CSF, IFN-γ, and MCP-1 during the early stages of infection with *E. coli* and *S. aureus*	[7,8,9]
Clavanin-MO		
Mastoparan-MO	Mastoparans can inhibit expression of Toll-like receptor 4 (TLR4), TNF-α, and interleukin-6 (IL-6) Mastoparan-MO can induce leukocyte migration to the site of infection in an in vivo model Treatment with Mastoparan-MO caused a decrease in proinflammatory cytokines IL-12, TNF-α, and IL-6	[10,11]

## Data Availability

The original contributions presented in the study are included in the article/supplementary material, further inquiries can be directed to the corresponding author/s.

## Supplementary Materials

The following supporting information can be downloaded at: https://www.mdpi.com/article/10.3390/ijms25136851/s1, Figure S1: Cytotoxicity controls.; Table S1: Primer sequences used for gene expression analyses by quantitative real-time PCR.
