# Supplementary material for: Screening of the Skin-Regenerative Potential of Antimicrobial Peptides: Clavanin A, Clavanin-MO, and Mastoparan-MO
Source: Int J Mol Sci. 2024 Jun 22;25(13):6851. doi: 10.3390/ijms25136851 (PMC11241485; doi:10.3390/ijms25136851)
Supplement: Supplementary file 1 [file ijms-25-06851-s001.zip › ijms-3015003-supplementary.pdf]

Table S1. Primer sequences used for gene expression analyses by quantitative real-time PCR.

Gene	Forward Primer	Reverse Primer
Fibroblast growth factor 2 (FGF2)	CAAGGACCCCAAGCGGCTGT	AGCTTGATGTGAGGGTCGCTCTT
Marker of proliferation Ki-67 (KI67)	TAACACCATCAGCAGGGAAAG	CTGCACTGGAGTTCCCATAAA
Elastin (ELN)	AAGGCTGCCAAGTACGGAGT	CAAACTGGGCGGCTTTGG
Matrix metalloproteinase 1 (MMP1)	GAGCTTCCTAGCTGGGATATTG	ACTGGCCTTTGTCTTCTTTCT
Hyaluronic acid synthase 2 (HAS2)	CTCGCAACACGTAACGCAAT	CAGTGCTCTGAAGGCTGTGT
C-X-C chemokine receptor type 4 (CXCR4)	CATCCTCATCCTGGCTTTCTT	CACACCCTTGCTTGATGATTTC
C-X-C chemokine receptor type 7 (CXCR7)	GTGGTGGTCTGGGTGAATATC	ATGTAGCAGTGCGTGTCATAG
B-cell lymphoma 2 protein (BCL2)	CAAAGCTGCAGGCTGTTTAAG	GTCTGTCTGTGTGTGTGATGT
Glyceraldehyde 3-phosphate dehydrogenase (GAPDH)	CCCTGGACATCGAGATCGC	TGTGCTCCTGCTTGGACTC

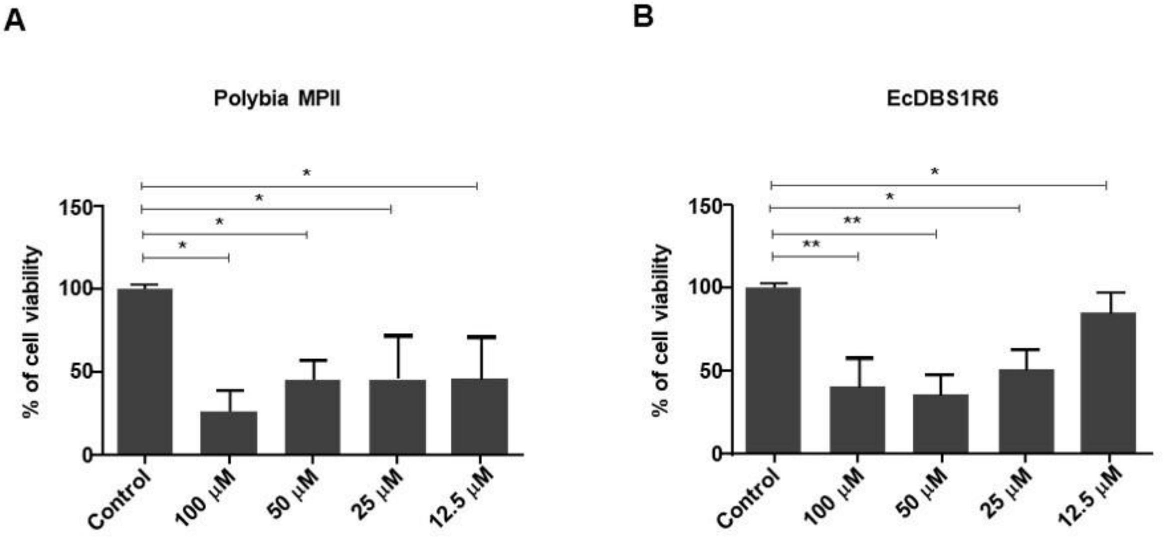

Figure S1. Cytotoxicity controls. Cytotoxic potential of the peptides Polybia-MPII and EcDBS1R6 were tested in Human primary dermal fibroblasts (hFibs) at concentrations of 100, 50, 25, and 12.5 µM. The mean ± SD of three independent experiments was plotted. The statistical analysis was carried out using a one-way ANOVA and Tukey posthoc tests. Significant differences regarding the control are marked with asterisks (*p<0.05; **p<0.01; ***p<0.001).
